# Effect of respiratory physiotherapy on symptom severity in clinical laryngopharyngeal reflux disease: A controlled study

**DOI:** 10.1007/s00405-025-09775-1

**Published:** 2025-10-15

**Authors:** Pavla Horova, Martin Dvoracek, Karol Zelenik, Jaromir Zatloukal, Tomas Rybnikar, Katerina Raisova

**Affiliations:** 1 Department of Physiotherapy, Faculty of Physical Culture, Palacký University Olomouc, Olomouc, 779 00 Czech Republic; 2 Department of Otorhinolaryngology and Head and Neck Surgery, Faculty of Medicine and University Hospital of Ostrava, Ostrava, 708 52 Czech Republic; 3 Department of Respiratory Medicine, University Hospital Olomouc, Olomouc, 779 00 Czech Republic; 4 Department of Otorhinolaryngology and Head and Neck Surgery, SPEA Olomouc s.r.o, Olomouc, Czech Republic

**Keywords:** Laryngopharyngeal reflux disease, Inspiratory muscle training, Diaphragmatic breathing, Reflux symptom index, Hull airway reflux questionnaire

## Abstract

**Purpose:**

Inspiratory muscle training (IMT) has demonstrated efficacy in improving symptoms and oesophageal sphincter function in patients with gastroesophageal reflux disease (GERD). However, evidence for its use in laryngopharyngeal reflux (LPR), a related but distinct condition, remains limited and inconclusive. We aim to assess the efficacy of an eight-week respiratory physiotherapy program – comprising IMT and diaphragmatic breathing – in reducing symptom severity in patients with clinical LPR.

**Methods:**

In this prospective, controlled clinical study, 37 patients with clinical LPR were allocated into three groups: a control group receiving dietary and pharmacological treatment and intervention groups receiving the same treatment supplemented with respiratory physiotherapy using either Threshold IMT or Airofit. Maximal inspiratory pressure (PImax) was used to evaluate inspiratory muscle strength. Symptom severity was measured using the Reflux Symptom Index (RSI) and Hull Airway Reflux Questionnaire (HARQ) at baseline and after 8 weeks.

**Results:**

Both experimental groups showed a significant increase in inspiratory muscle strength (36.3% for Airofit, *p* = 0.005; 38.3% for Threshold IMT, *p* < 0.001), unlike the control group (*p* = 0.260). RSI scores decreased significantly in all groups but was more pronounced in the intervention groups compared to the control group (*p* < 0.05). HARQ scores improved across all groups without any significant between-group difference.

**Conclusions:**

Standard care supplemented with respiratory physiotherapy integrating IMT significantly reduced symptom severity in LPR. Both the Threshold IMT and Airofit devices proved effective. These findings support respiratory physiotherapy as a feasible, non-pharmacological therapeutic option in LPR management.

## Introduction

Laryngopharyngeal reflux (LPR), also termed airway reflux or extraoesophageal reflux disease, is a frequently encountered condition in otolaryngological practice, with a reported prevalence of approximately 23% among patients presenting to ENT clinics in Europe [1]. LPR is characterized by the retrograde flow of gastroduodenal contents beyond the upper oesophageal sphincter (UES), leading to irritation or damage of the respiratory tract mucosa [2]. Given the heightened sensitivity of the airway tissues [3], even episodes of minimal reflux can elicit significant clinical manifestations. These include chronic cough, hoarseness, throat tickling, dysphagia, globus pharyngeus, sour taste, otitis media, and accelerated dental erosion [4].

The standard management of LPR typically includes anti-reflux dietary modifications, lifestyle interventions, and pharmacological therapy, such as treatment with proton pump inhibitors (PPIs) [5]. However, current evidence indicates that a substantial proportion of patients experience suboptimal symptom control with PPI monotherapy [6], underscoring the need for the development and evaluation of adjunctive or alternative non-pharmacological treatment strategies to enhance therapeutic outcomes in individuals with refractory LPR.

Aerosol reflux is often the primary mechanism underlying LPR and is associated with transient lower oesophageal sphincter relaxations (tLESRs) and UES relaxation [2]. During tLESRs, there is a coordinated relaxation in the lower oesophageal sphincter (LES) and a simultaneous reduction in crural diaphragmatic tension—both critical components of the anti-reflux barrier [7, 8]. Notably, given that the diaphragm accounts for up to 85% of the total contractile force of the barrier [9, 10] and is a striated muscle that is partially under voluntary control, increasing LES pressure by strengthening the diaphragm through inspiratory muscle training (IMT) [11, 12] or postural training [13] can be a promising non-pharmacological strategy for enhancing anti-reflux defence. A systematic review summarising studies investigating the effect of respiratory training in patients with gastroesophageal reflux disease (GERD) reported beneficial effects on both the occurrence and intensity of symptoms following respiratory training, including an increased LES pressure and reduced frequency and duration of tLESRs [14]. Therefore, we hypothesise that therapy aimed at strengthening the diaphragm could represent an effective non-pharmacological intervention for patients with LPR as well.

The primary aim of this study was to investigate the efficacy of respiratory physiotherapy in alleviating symptoms in patients with LPR. The secondary aims included assessing changes in inspiratory muscle strength and comparing the efficacy of a novel electronic training device (Airofit) to that of a conventional device (Threshold IMT).

## Materials and methods

### Patient involvement, inclusion/exclusion criteria, and basic examination

Patients with clinical LPR who were referred from the ENT department were considered for the study. Inclusion criteria included patients aged 18 years or older with symptomatic LPR, confirmed with laryngoscopy examination. Patients who met the inclusion criteria were asked about personal history, course of LPR, previously underwent examinations, and pharmacological treatment. Exclusion criteria included history of fundoplication surgery, acute respiratory infection within the last 4 weeks, chronic respiratory diseases, decompensated cardiopulmonary disease, active smoking, alcohol abuse, and pregnancy. Moreover, only patient with physiologic spirometry results were included in the study.

Patients who met the criteria and provided written informed consent were included in the study. They were divided into Experimental group 1 (“Threshold IMT group”), Experimental group 2 (“Airofit group”), and Control group. Group allocation was conducted through stratification based on age and sex to ensure group comparability. At the outset of the study, only the Threshold IMT device was available. Once the Airofit system became available, eligible patients were assigned to the Airofit group as well, while concurrent recruitment into the Threshold IMT group continued. Consequently, the final sample sizes differ across groups.

**Fig. 1:**
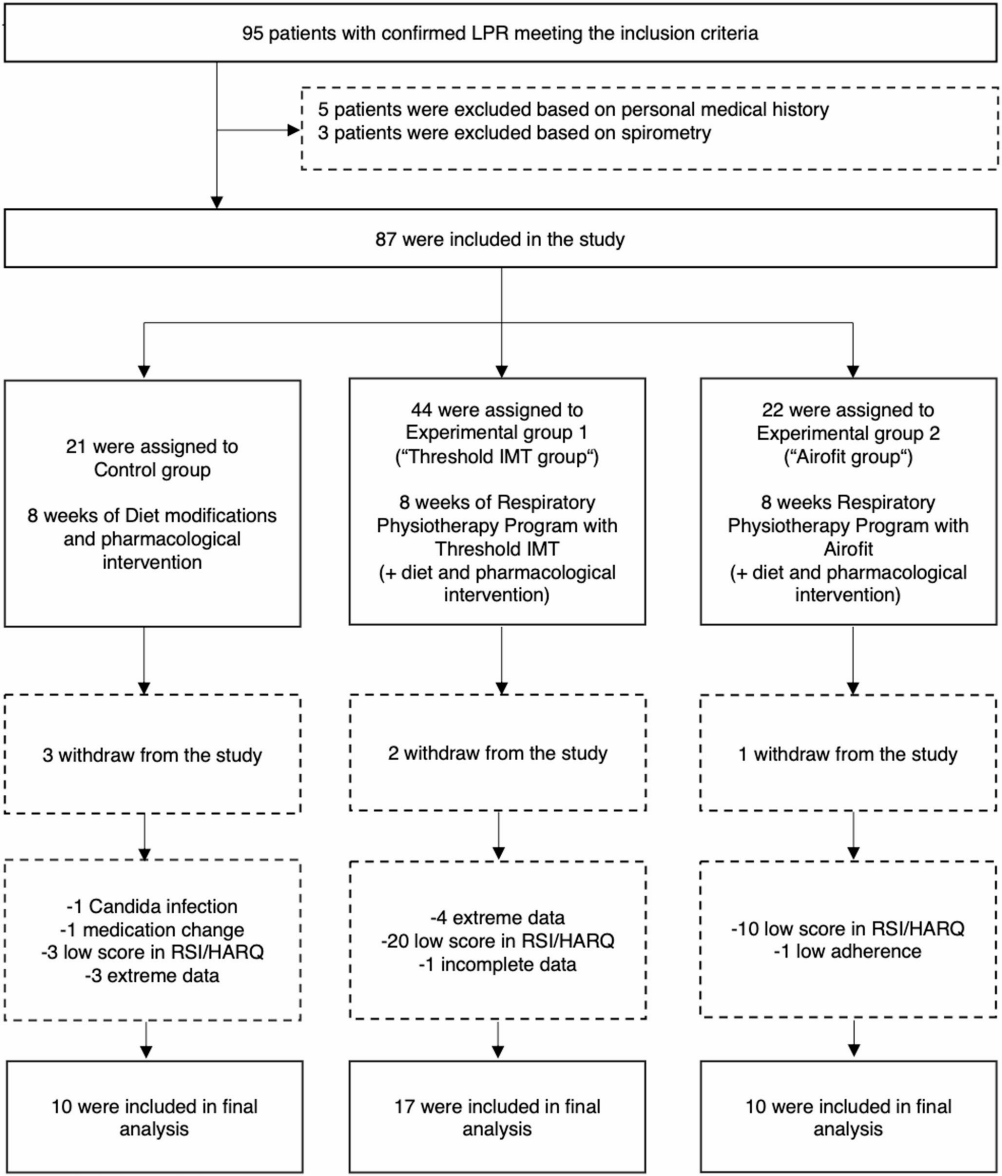
Flowchart of research process

All patients underwent an initial examination that included symptom assessment with questionnaires and an evaluation of respiratory muscle strength. After 8 weeks, all patients underwent a final examination, which was identical to the initial assessment. The study procedure is illustrated in Fig. 1.

### Symptom assessment

Symptom severity was assessed with the Reflux Symptom Index (RSI) and the Hull Airway Reflux Questionnaire (HARQ) at baseline and after an eight-week program; the total score of each questionnaire was used. The RSI [15] and HARQ [16] are self-administered questionnaires consisting of 9 and 14 items, respectively. Patients were asked to score the intensity of their symptoms in the last two weeks on a Likert scale from 0 (no problem) to 5 (severe/frequent problem). The threshold positivity of both questionnaires is 13 points [15, 16].

### Inspiratory muscle strength assessment

Inspiratory muscle strength was assessed with the Geratherm Respiratory Diffustik (GmbH; Bad Kissingen, Germany) device at baseline and after the eight-week program. The patient was placed in an upright sitting position with lower extremity support, and the nasal airways were closed with a clip. Before the assessment, the course of the examination was explained to the patient. Then, the patient was instructed to perform six tidal breaths, followed by a maximal expiration to residual volume and then a maximal inspiratory effort against an occluded valve (Müller manoeuvre). Five repetitions were performed, with periods of tidal breathing between attempts. The highest value from three acceptable manoeuvres (with variability < 10%) was used for analysis. The assessment was performed according to the European Respiratory Society statement on respiratory muscle testing [17].

### Dietary modifications, pharmacological treatment, and respiratory physiotherapy programs

All patients included in the study were familiarized with the dietary modifications during the initial assessment and were instructed to adhere to their prescribed pharmacological treatment, specifically the intake of PPIs twice daily, for the following 8 weeks and to record any violations. Patients were asked to avoid the consumption of acidic, fatty and spicy foods, sparkling drinks, alcohol, coffee, sweets, and leavened pastry during the eight-week period. Dietary modifications were based on recommendations by Lechien et al. [18] and provided to patients in the form of an information leaflet. This leaflet also included a list of acidic foods to avoid and recommended alkaline foods, sorted by pH value according to Jamie Koufman [19].

Aside from the dietary modifications and pharmacological treatment, patients enrolled in the experimental groups underwent an eight-week respiratory physiotherapy program including diaphragmatic breathing training (front, side, and back) and IMT with a resistive breathing device (Threshold IMT or Airofit device based on the experimental group). Patients performed three sets of 10 breaths twice daily in three different postural positions: supine position with the lower limbs in 90 degrees of hip and knee flexion, Brügger sitting, and quadruped position. Once a week (8 sessions in total), patients attended supervised physiotherapy sessions during which the physiotherapist monitored the correct performance of diaphragmatic breathing and IMT and adjusted the training resistance individually according to each patient’s progression and tolerance. The program was applied uniformly to all patients according to the same predefined exercise protocol.

#### Threshold IMT

Patients included in Experimental group 1 (“Threshold IMT group”) performed IMT with Threshold IMT. This device operates on a pressure-threshold valve mechanism, in which resistance is influenced by the stiffness of an internal spring. To initiate airflow, the patient must therefore exert a force corresponding to the preset resistance, which can be set in the range of 9–41 cmH_2_O.

The initial resistance was set at 30% PImax and was progressively increased by 2 cmH_2_O each week until the maximum resistance of the device was reached. The protocol for Experimental group 1 was based on that from previous studies that demonstrated the effect of IMT in patients with GERD [11, 12].

#### Airofit

Patients included in Experimental group 2 (“Airofit group”) performed IMT with the Airofit device, a novel electronic breathing trainer based on air flow resistance, which is determined by the diameter of the space and the speed of the patient’s breath. The device is compatible with a mobile feedback application and expert training module, allowing the physiotherapist to monitor training performance and adjust resistance levels accordingly. Patients are required to generate sufficient inspiratory force to reach the target resistance, which is displayed in the mobile application. Compared to Threshold IMT, Airofit offers unrestricted maximum resistance settings and allows for objective verification of training adherence and effort.

The initial resistance was set at 50% PImax and was increased weekly according to the patient’s current PImax, which was measured at the beginning of each supervised session with the physiotherapist. The protocol for Experimental group 2 was based on the findings of Langer et al. [20], which showed better outcomes at a resistance of 50% PImax using an air flow resistance breathing device.

### Statistical analysis

The collected data were anonymised and processed using Microsoft Excel (Office 365). Statistical analyses were conducted with IBM SPSS Statistics 28.01.1.14 (IBM, NY, USA). Inspiratory muscle strength was measured in kilopascals (kPa) and then converted to centimetres of water (cmH₂O) and expressed as a percentage of the normative value according to Evans and Whitelaw [21], allowing normalization of respiratory muscle strength for all participants regardless of gender.

Descriptive statistics are presented as the arithmetic mean (M), median (med), standard deviation (SD) and interquartile range (IQR). Within-group differences were determined using the Wilcoxon signed-rank test, and between-group differences were assessed using the Mann–Whitney U test. A significance level of α = 0.05 was applied for all statistical tests.

## Results

### Participant demographics

Thirty-seven participants completed the study and were enrolled in the final analysis, including 28 women and 9 men. The mean age was 46.86 years (SD 12.7), and the mean BMI was 25.48 kg/m² (SD 4.7). Figure 1 presents the participant recruitment flow, and Table 1 summarizes the baseline demographic and clinical characteristics of the study population.

**Table 1:** Participant baseline characteristics

Baseline characteristics	*N* = 37
Mean age (SD), years	46.86 (12.7)
Sex, women: men	28:9
Mean BMI (SD), kg.m^−2^	25.48 (4.7)
Mean FEV_1_ (SD), % predicted	99.84 (10.3)
Mean PImax (SD), % predicted	74.74 (25.3)
Med RSI (IQR), total score	19 (9)
Med HARQ (IQR), total score	28 (13)

*BMI *body mass index, *FEV*_*1*_ forced expiratory volume in 1 s, *PImax* pressure inspiratory maximum, *RSI* Reflux Symptom Index, *HARQ* Hull Airway Reflux Questionnaire

### Reflux symptom severity

The pre- and post-treatment differences in reflux symptom severity are presented in Table 2. After the intervention, all groups showed a significant reduction in RSI scores. The control group improved by 6.5 points (*p* = 0.005), while the Airofit and Threshold IMT groups showed greater improvements of 10.5 and 11.0 points, respectively (*p* = 0.005, *p* < 0.001). The reduction in RSI was significantly greater in both experimental groups compared to the control group (Airofit: *p* = 0.043; Threshold IMT: *p* = 0.011).

**Table 2:** Pre- and post-treatment differences in reflux symptom severity

	*N*	Pre- (med, IQR)	Post- (med, IQR)	MD	*p* (within)	*P* (between)
**RSI, total score**						
Control group	10	21.0 (10)	14.5 (11)	6.5	0.005*	-
Threshold IMT group	17	19.0 (6)	8.0 (9)	11.0	<0.001*	0.011**
Airofit group	10	20.0 (10)	9.5 (8)	10.5	0.005*	0.043**
**HARQ, total score**						
Control group	10	28.0 (13)	23.5 (19)	4.5	0.022*	-
Threshold IMT group	17	26.0 (17)	11.0 (13)	15.0	<0.001*	0.059
Airofit group	10	32.0 (10)	15.5 (16)	16.5	0.005*	0.529

*RSI *reflux symptom index, *HARQ *Hull Airway Reflux Questionnaire, *med *median, *IQR *interquartile range, *MD* mean difference, *N* population

*Wilcoxon signed-rank test, **Mann–Whitney U test, Significance was set at *p < *0.05

HARQ scores also decreased significantly across all groups. The control group showed an improvement of 4.5 points (*p* = 0.022), the Airofit group of 16.5 points (*p* = 0.005), and the Threshold IMT group of 15.0 points (*p* < 0.001). However, between-group differences in HARQ improvement were not statistically significant.

### Inspiratory muscle strength

In all patients included in the final analysis, the average pre-treatment inspiratory muscle strength was 74,74% of the predicted value, indicating weakness of inspiratory muscles; 51.4% (*n* = 19) exhibited inspiratory muscle strength below 80% of the predicted value, 32.4% (*n* = 12) had values within the 80–100% range, and 16.2% (*n* = 6) exceeded 100% of the predicted value. No statistically significant difference was found between the experimental and control groups in pre-treatment inspiratory muscle strength.

**Fig. 2:**
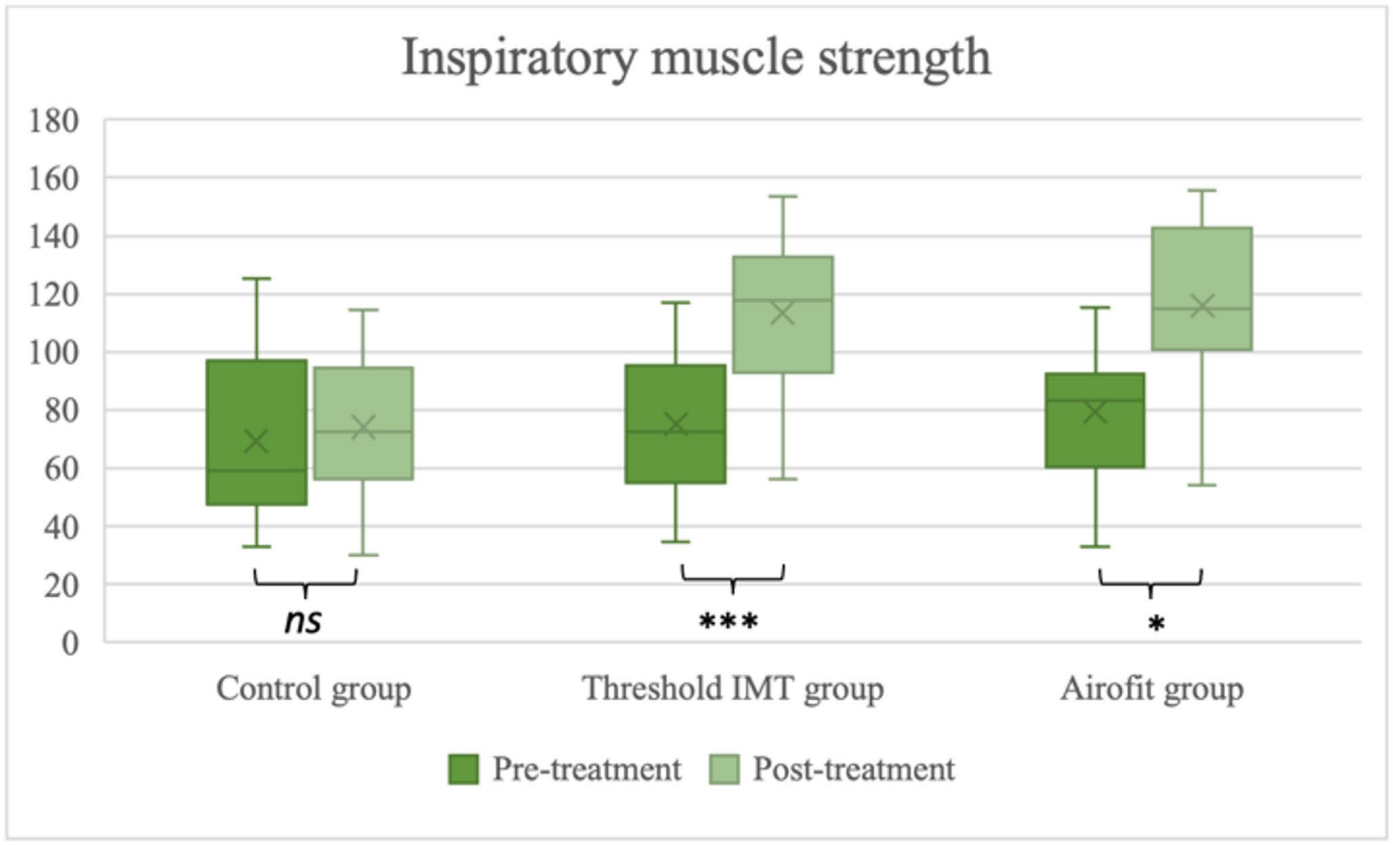
Pre- and post-treatment comparison of the inspiratory muscle strength across the groups. ns, not significant; *p < 0.05; **p < 0.005; ***p < 0.001

In both experimental groups, a statistically significant increase in inspiratory muscle strength was observed after 8 weeks of IMT—by an average of 36.3% (*p* = 0.005) of the predicted value in the group training with the Airofit device and by 38.3% (*p* < 0.001) in the group using Threshold IMT. The change in inspiratory muscle strength in the control group was not statistically significant (MD = 4.73% of the predicted value, *p* = 0.260). Patients in the experimental groups achieved significantly higher inspiratory muscle strength after 8 weeks of training compared to patients in the control group (*p* = 0.009 and *p* = 0.002, respectively). The results are illustrated in Fig. 2.

## Discussion

To our knowledge, this is the first study to evaluate whether a respiratory physiotherapy program, focused on strengthening the inspiratory muscles and activating the diaphragm with resistance devices, can influence the severity of symptoms in patients with clinical LPR. Our aim was to determine whether combining IMT with diaphragmatic breathing and postural training could offer an effective adjunctive therapy to standard treatment approaches.

The results of our study indicate that an eight-week intervention consisting of diaphragmatic breathing and IMT in specific postural positions using either the Threshold IMT or Airofit device led to a significant reduction in symptom severity as measured by the RSI. Participants who followed dietary modifications and pharmacological treatment alone showed only minor improvements, with RSI scores remaining above the diagnostic threshold. In contrast, patients who combined dietary modifications and pharmacological treatment with respiratory physiotherapy achieved an average reduction in RSI scores of up to five points below the positivity cutoff. These findings suggest that the addition of IMT, diaphragmatic breathing, and specific postural positions may enhance the effectiveness of standard pharmacological and dietary interventions in patients with clinical LPR.

Symptom severity was also assessed using the HARQ, which is particularly sensitive to chronic cough and LPR-related symptoms. This tool, developed by Morice et al. [16], is grounded in the concept of airway reflux as a key driver of cough hypersensitivity syndrome, often initiated by micro aspiration of refluxate into the airways. In the present study, both the intervention and control groups showed a significant reduction in HARQ scores after eight weeks. However, no statistically significant difference was observed between the groups. This finding may primarily reflect the limited sample size, which reduced the statistical power to detect significant changes in cough-related symptoms. Further studies with larger sample sizes or extended treatment durations are warranted to explore this relationship.

Airofit employs a different resistance mechanism to Threshold IMT and provides biofeedback through smartphone connectivity. It also allows individualized resistance settings and monitoring of adherence, which may improve therapy compliance [22]. Despite its technological differences, the results showed that patients training with Airofit achieved comparable improvements in inspiratory muscle strength and reductions in LPR symptoms, as measured by the RSI, to those observed in patients using Threshold IMT. These findings suggest that Airofit may represent a suitable alternative for patients with inspiratory muscle weakness and LPR symptoms, particularly due to its potential to enhance patient motivation and enable remote monitoring of therapy.

Our findings in patients with clinical LPR are consistent with previous studies demonstrating the beneficial effects of respiratory physiotherapy in individuals with GERD. Eherer et al. [23] showed that abdominal breathing significantly alleviated symptoms in patients with GERD, highlighting the importance of diaphragmatic activation and restoration of a physiological breathing pattern. The success of their program indicates that even non-device-based breathing retraining can be effective, and that breath pattern correction is essential in these patients. Similar conclusions were reached by Ahmadi et al. [24], who demonstrated that regular diaphragmatic breathing performed five times daily over four weeks significantly increased lower esophageal sphincter (LES) pressure and improved quality of life in patients with reflux. Notably, diaphragmatic breathing was more effective than aerobic exercise in improving patient-reported outcomes, suggesting its utility even in patients unable to engage in physical activity. Also, Sun et al. [25] found that long-term diaphragmatic biofeedback training enhanced gastroesophageal junction pressure and cardiac diaphragmatic tone, while also reducing the need for acid-suppressive therapy. These findings support the hypothesis that breathing pattern retraining may improve antireflux barrier function and contribute to sustained symptom relief in reflux disorders. Taken together with previous evidence, our results reinforce the therapeutic potential of diaphragmatic breathing as a core component of multimodal respiratory physiotherapy in the management of reflux-related disorders, including LPR.

The IMT protocol using the Threshold IMT device was based on the study from De Miranda Chaves et al. [12], who demonstrated that an 8-week IMT program starting at 30% resistance resulted in a significant increase in inspiratory strength in patients with GERD. Our findings confirm a similar effect in patients with clinical LPR, with a concurrent reduction in symptom intensity. Nobre e Souza et al. [11] investigated a progressive IMT protocol, in which the training load was adjusted throughout the program—similarly to our study. They concluded that the intervention led not only to reduced symptom intensity and heartburn, and increased pressure at the esophagogastric junction, but also to a decrease in the number and duration of tLESRs. These changes suggest a mechanical improvement in sphincter function, which may also explain the potential mechanism of IMT effectiveness in patients with LPR, where tLESRs are considered a key contributor to sphincter dysfunction.

We identified two studies that specifically evaluated the effect of breathing exercises on extraoesophageal symptoms, including LPR. Moffa et al. [26] proposed a modified breathing training protocol that combined diaphragmatic breathing with controlled swallowing. Their intervention resulted in rapid symptom improvement according to the RSI, as well as better laryngoscopy findings assessed by the Reflux Finding Score (RFS). Barillari et al. [27] also reported significant improvements in both RSI and RFS in patients with LPR confirmed by multichannel intraluminal impedance-pH (MII-pH) monitoring after a three-month voice therapy program. Their intervention combined voice therapy, diaphragmatic breathing, posture control and relaxation training, with diet modifications and pharmacological treatment. Our results, demonstrating that breathing training can reduce LPR symptom severity, are therefore in agreement with these findings. Moreover, our study introduces another alternative respiratory training strategies that may be applicable and beneficial for this patient population.

The findings of the present study support the clinical integration of respiratory physiotherapy as an additional therapeutic option alongside dietary modifications and pharmacological treatment for patients with clinical LPR. Due to its affordability, favourable safety profile and minimal side effects, IMT could be used as an accessible intervention in both primary and specialist care settings. Furthermore, it can be implemented without the need for extensive equipment or resources, making it suitable for outpatient physical therapy programmes and home rehabilitation. Future studies should determine whether this type of treatment could be used as a first-line therapy or whether it could effectively treat patients who do not respond adequately to pharmacological treatment.

This study, indicating positive effect of IMT on LPR symptoms, has several limitations. The relatively small sample size may have limited the statistical power to detect between-group differences, particularly for HARQ outcomes. In addition, group assignment was influenced by the current availability of intervention devices, which could have led to selection bias.

Furthermore, no instrumental outcome measures (e.g., Reflux Finding Score, impedance-pH monitoring, or oesophageal manometry) were used to verify objective changes.

Further research is required to evaluate these factors and explore how device characteristics influence long-term adherence, therapeutic effectiveness, and patient satisfaction. These limitations should be considered when interpreting the results and addressed in future research to strengthen the evidence base. It would also be beneficial to assess the durability of treatment effects and to investigate the mechanisms by which inspiratory muscle function may influence UES competence.

## Conclusions

The respiratory physiotherapy program, which consisted of IMT combined with diaphragmatic breathing, significantly reduced LPR symptoms when added to standard dietary and pharmacological treatment. The Airofit device proved equally effective as the Threshold IMT and offered practical advantages. These findings support the inclusion of respiratory physiotherapy as a safe, low-cost adjunctive therapy for patients with LPR. Further research with larger sample populations and objective measures is warranted.

## Data Availability

The data presented in this study are stored in the Palacký University Olomouc OneDrive and are available upon request from the corresponding author (Pavla Horová, MSc - email: pavla.horova01@upol.cz).
